# Associations between lifetime reproductive events among postmenopausal women with bipolar disorder

**DOI:** 10.1007/s00737-024-01533-2

**Published:** 2024-11-14

**Authors:** Katherine Gordon-Smith, Amy Perry, Arianna Di Florio, Nicholas Craddock, Ian Jones, Lisa Jones

**Affiliations:** 1 Psychological Medicine, University of Worcester, Worcester, UK; 2 Division of Psychological Medicine and Clinical Neurosciences, Cardiff University, Cardiff, UK; 3 Three Counties Medical School, University of Worcester, Severn Campus, Hylton Road, Worcester, WR2 5JN UK

**Keywords:** Bipolar disorder, Women, Lifetime reproductive events, Perimenopause

## Abstract

**Purpose:**

The premenstrual phase of the menstrual cycle, childbirth and perimenopause often coincide with a worsening of mood symptoms in women with bipolar disorder (BD). To date, findings from the limited number of studies investigating associations between these events among women with BD have been inconsistent. This study aimed to investigate associations between episodes in relation to the perimenopause and (i) premenstrual symptoms and (ii) postpartum mood episodes in a large sample of postmenopausal women with BD.

**Methods:**

Among 567 postmenopausal women with BD, recruited as part of the UK Bipolar Disorder Research Network, relationships between reproductive event-associated mood symptoms/episodes were examined. Multivariate binary analyses were carried out to identify if history of premenstrual symptoms and/or postpartum episodes predicted the occurrence of mood episodes in relation to the perimenopause, controlling for potential confounders including number of mood episodes per illness year.

**Results:**

History of premenstrual symptoms was associated with experiencing any type of mood episode, and depression specifically, during the perimenopause (OR 6.189, *p* < 0.001 and OR 2.709, *p* = 0.019 respectively). History of postpartum depression within 6 weeks of delivery was associated with depressive episodes during the perimenopause (OR 2.635, *p* = 0.027). Postpartum mania was not a significant predictor.

**Conclusions:**

Our findings suggest that women with BD with a history of premenstrual symptoms and postpartum depression are potentially at increased risk of experiencing episodes of depression in relation to the perimenopause. There are clinical and self-management implications in identifying a subgroup of women with BD who may be particularly vulnerable to episodes of mood disturbance during reproductive events.

## Article highlights

Relationships between reproductive events investigated among 567 postmenopausal women with bipolar disorder.

History of premenstrual symptoms and postpartum depression was associated with episodes of depression during the perimenopause.

No significant relationships were identified with (hypo)mania in relation to the perimenopause.

A subgroup of women with bipolar disorder may be particularly vulnerable to episodes of mood disturbance during reproductive events.

## Introduction

Previous research has shown that key events in the female reproductive lifecycle (premenstrual, perinatal and perimenopausal period) often coincide with an exacerbation of mood symptoms in women with bipolar disorder (BD). One study of 158 women with BD found that up to 77% reported worsening of symptoms associated with at least one reproductive event (Perich et al. 2017a). The association with the perinatal period has been studied in depth, with a meta-analysis of 37 studies investigating the risk of postpartum recurrence in women with BD finding an overall risk of 37% (Wesseloo et al. 2016). Much research in this field has focused on the prevalence of postpartum psychosis, which is considerably higher in women with BD (23%) (Perry et al. 2021) than the general population (0.89 to 2.6 in 1000 women) (VanderKruik et al. 2017).

There has also been growing interest in the occurrence of premenstrual syndrome (PMS) and premenstrual dysphoric disorder (PMDD) in BD. PMS is characterised by affective, behavioural, cognitive and physical symptoms during the late luteal phase of the menstrual cycle which end at, or within a few days after, menstruation onset. A meta-analysis reviewing epidemiological studies reported a general population pooled prevalence rate of 47.8% for PMS worldwide (Direkvand-Moghadam et al. 2014). Reported rates among women with BD vary, with the findings of a review highlighting that retrospective studies indicate that 25–77% of women with BD report PMS (Teatero et al. 2014). PMDD is less common than PMS and characterised by more severe affective, psychological and physical symptoms also occurring in the luteal phase of the menstrual cycle. PMDD became an official diagnosis under the Depression and Depressive Disorders chapter in the fifth edition of the DSM-5 (American Psychiatric Association 2013). DSM-5 diagnostic criteria for PMDD require the presence of at least five symptoms, one being mood related, with symptoms resulting in functional impairment. In a recent meta-analysis of 44 studies consisting of 50,659 participants, the pooled general population prevalence was 3.2% for a confirmed diagnosis of PMDD (requiring prospective monitoring of symptoms over two cycles) and 7.7% for a provisional diagnosis (Reilly et al. 2024). Among women with BD, a systematic review found the proportion who reported symptoms consistent with a provisional diagnosis of PMDD ranged from 27 to 76% (Sharma et al. 2022). This suggests PMDD is more common among women with BD than in the general population. However, none of the studies included in the review confirmed the provisional diagnosis of PMDD using prospective daily ratings.

While the relationship between BD and the perinatal period in particular is well established, there is a lack of research examining the occurrence of mood episodes and symptoms in women with BD in relation to the perimenopause. A recent UK study on over 100,000 women has found that the risk of a first occurrence of mania in the perimenopause is over 3 times that of the late reproductive years (Shitomi-Jones et al. 2024). For women with pre-existing BD, a systematic review identified that the perimenopause could be a time of increased vulnerability (Perich et al. 2017b). The review included 9 studies (3 longitudinal and 6 cross-sectional) and a total of 273 women with BD. The menopause was found to be associated with increased symptoms overall and with depression in particular being the most common mood symptoms experienced. Specifically focusing on mood episodes, Marsh et al. (2008) found among 47 perimenopausal aged women with BD, 68% experienced at least one depressive episode over a longitudinal monitoring period of on average 17 months and that episode frequency was significantly increased during the perimenopause compared to during women’s reproductive years.

The findings of a recent review suggest that a subset of women with BD may be particularly vulnerable to the impact of menstrual cycle events (Aragno et al. 2022) Further research investigating associations between reproductive events in women with BD support this suggestion. Among 158 women with BD, those who reported they had experienced postnatal episodes were significantly more likely to report experiencing worse mood symptoms in the premenstrual and menopausal period (Perich et al. 2017a). The authors indicated their findings suggested some women with BD have a greater vulnerability to episodes of mood disturbance in relation to reproductive events. Furthermore, a case series of five women with BD type I (BDI), highlighted a potential link between postpartum and menopausal mood episodes (Robertson Blackmore et al. 2008). All five women had experienced an episode of postpartum psychosis and an episode of mania around the time of the menopause and the majority did not experience mania or other mood episodes outside of these periods. In contrast, among 197 women with BDI, Payne et al. (2007) did not find any significant associations between reproductive cycle-associated mood symptoms, with premenstrual and postpartum mood symptoms not predicting perimenopausal mood symptoms. Marsh et al. (2015) also found that among 44 women with BD, self-reported history of mood exacerbation premenstrually and/or during the postpartum was not significantly associated with mood symptom severity during the perimenopause.

To date the findings of the limited number of studies investigating associations between reproductive events among women with BD have been inconsistent. Studies involving women who have experienced or were experiencing the perimenopause have included small sample sizes, limiting the ability to stratify by episode types during this period. In a large sample of postmenopausal women with BD, this study aimed to investigate associations between both (i) premenstrual mood symptoms and (ii) postpartum mood episodes and episodes in relation to the perimenopause, stratifying by episode type in the postpartum and perimenopause periods.

## Methods

This study was part of an on-going research programme into the genetic and non-genetic aetiology of BD (Bipolar Disorder Research Network, bdrn.org). The research programme has UK National Health Service (NHS) Health Research Authority (HRA) approval – Research Ethics Committee (REC) reference (MREC/97/7/01) and local approvals in all participating NHS Trusts/Health Boards.

### Recruitment of participants

Participants were recruited into the research programme from across the UK using both systematic and non-systematic recruitment methods. Participants were recruited systematically via National Health Service (NHS) psychiatric services (community mental health teams and lithium clinics). Non-systematic recruitment methods included advertising for volunteers using local and national media and through UK-based charities for individuals with BD, for example Bipolar UK.

The research programme inclusion criteria were (i) DSM-IV/DSM-5 diagnosis of bipolar disorder (bipolar disorder type I, bipolar disorder type II, schizoaffective disorder bipolar type or bipolar disorder not otherwise specified) (American Psychiatric Association 2000, 2013), (ii) aged at least 18 years, (iii) mood symptoms to have started before the age of 65 years, (iv) able to provide written informed consent, and (v) of European ancestry due to the focus of the research programme on genetic risk factors. Potential participants were excluded if they: (i) had only experienced affective illness in relation to, or as a consequence of, alcohol or substance abuse or dependence; (ii) had only experienced affective illness as a consequence of medical illness or medication; (iii) were biologically related to another study participant.

This study was carried out using a subset of women in the BDRN research programme who were postmenopausal and had provided information about mood episodes in relation to the perimenopause, the postpartum period and premenstrual symptoms (*n* = 567).

### Psychiatric assessment

A modified version of the semi-structured Schedule for Assessment in Neuropsychiatry (SCAN) interview was used to collect detailed information on lifetime psychopathology (Wing et al. 1990). In most cases, participants’ psychiatric and medical case notes were also accessed to corroborate information given during the interview. Diagnoses and lifetime-ever clinical ratings including age at illness onset and number of lifetime illness episodes were made using all available clinical data. All interviews and diagnostic and rating procedures were carried out by trained psychologists and psychiatrists. In cases where there was doubt, ratings were made by at least two members of the research team blind to one another’s ratings and consensus was reached by discussion where necessary. Inter-rater reliability was formally assessed using 20 cases and was found to be high. The mean kappa score for DSM diagnosis was 0.84 and those for categorical data ranged from 0.81 to 0.97. Mean intra-class correlation coefficients for continuous data ranged from 0.92 to 0.99.

### Lifetime reproductive events

Data regarding the menopause were collected using a self-report questionnaire designed by the authors, either at the time of the initial clinical interview or subsequently as part of a questionnaire mail out sent to all BDRN participants in current contact with the research programme at that time (just under 4000 participants). Women who self-reported being postmenopausal were asked to report: (i) the age they went through the menopause; (ii) if they had experienced mood episodes related to the menopause; and, (iii) written details about any episodes. A pragmatic decision to ask postmenopausal women only was made due to the self-report method of data collection and women potentially being unsure if they were in the early stages of the perimenopause. The details provided were used to classify the episodes described creating a broad category of episode of any type (including unspecified mood episodes) and narrower definitions of episodes of depression and (hypo)mania. The narrower definitions of depression and (hypo)mania were rated where sufficient information was provided to be able to classify the polarity of the episode(s). The ratings were made based on the most significant episode reported during the perimenopause which was rated hierarchically according to the written descriptions of the episodes and symptoms. In cases where a woman reported an episode of mania requiring hospitalisation and/or psychosis and also described an episode of depression, the episode of mania was rated hierarchically as the most impairing episode. These ratings were made by at least two members of the research team blind to one another’s ratings and consensus was reached by discussion where necessary. At the same time the Premenstrual Symptoms Screening Tool (PSST)(Steiner et al. 2003) was used to ask women to rate their past usual experience of 14 premenstrual syndrome symptoms and the resultant level of occupational and social impairment, during their lifetime, using a four-point scale: not at all, mild, moderate or severe. The responses were used to make a broad and narrow definition of symptoms suggestive of moderate to severe premenstrual syndrome (PMS) and premenstrual dysphoric disorder (PMDD) respectively according to the PSST tool’s criteria. For possible PMDD this included at least one of four core symptoms rated severe, at least four additional symptoms “moderate to severe” and at least one of five domains of functioning rated severe. At interview, parous women (*n* = 432) provided information about the lifetime occurrence of mood episodes in each perinatal period. Case note data were used to corroborate the information provided about perinatal mood episodes. Interview and case note data were combined to rate the time of onset in weeks following delivery and polarity of the worst mood episode in each perinatal period. If this information could not be established at interview or from case note data a rating of unknown was made.

### Analysis

SPSS version 29.0 was used to analyse the data. Associations between the occurrence of menopausal episodes and (i) PMS/PMDD and (ii) postpartum mood episodes were initially explored using chi squared tests.

Three separate binary logistic regressions, using the enter method, were used to determine whether a history of postpartum episodes and premenstrual symptoms could predict the occurrence of mood episodes in relation to the perimenopause ((i) of any type, (ii) depression, (iii) (hypo)mania) after controlling for potential confounders: current age, number of mood episodes per illness year, parity (number of deliveries), age of onset of BD and reported age at menopause.

## Results

### Sample

The sample of 567 women is described in Table 1. Women had a median age of 59 years, 23.1% had been recruited systematically, 40.9% had completed higher education and 91.2% had lived as married. All women were of European ancestry due to the focus of the research programme on genetic risk factors with 94% of UK White ethnicity. The majority had a DSM diagnosis of bipolar I disorder (72.1%) or bipolar II disorder (22.4%). A further 2.6% had a diagnosis of schizoaffective disorder bipolar type and 2.8% bipolar disorder not otherwise specified. The median age of onset of bipolar disorder was 22.5 years. The median number of mood episodes (of any polarity), depressive episodes and hypo(manic) episodes per illness year respectively was 0.51, 0.29 and 0.21. Median age at menopause was 50 years.

**Table 1:** Demographic and clinical characteristics of sample of postmenopausal women with bipolar disorder (*n* = 567^)

Demographic	
Current age, years, median (IQR)	59 (10)
Systematically recruited, % (n)	23.1 (127/550)
Highest educational level (higher education), % (n)	40.9 (218/533)
Marital history (has married), % (n)	91.2 (495/543)
Ethnicity (UK White), % (n)	94.4 (535/567)
**Clinical**	
Bipolar disorder type I, % (n)	72.1 (409/567)
Bipolar disorder type II, % (n)	22.4 (127/567)
Schizoaffective disorder bipolar type, % (n)	2.6 (15/567)
Bipolar disorder not otherwise specified, % (n)	2.8 (16/567)
Age at illness onset, years, median (IQR)	22.5 (13)
Average number of mood episodes (of any polarity) per illness year, median (IQR)	0.51 (0.66)
Average number of depressive episodes per illness year, median (IQR)	0.29 (0.38)
Average number of hypo(manic) episodes per illness year, median (IQR)	0.21 (0.33)
Age at menopause, years, median (IQR)	50 (7)

IQR, interquartile range

^ denominators vary due to missing/unknown data

### Rates of reproductive event-associated mood symptoms/episodes

359 out of 567 postmenopausal women were able to report the definite absence or presence of a mood episode in relation to the perimenopause, with almost two thirds of those (58.5%) experiencing an episode (Table 2). 21.4% and 8.9% of women reported an episode of depression and (hypo)mania in relation to the perimenopause respectively. 51.3% and 22.7% of women retrospectively reported symptoms suggestive of PMS and possible PMDD. Among parous women, 49.5% experienced a mood episode of any type within 6 weeks postpartum, 26.4% and 19.6% had experienced an episode of depression and mania within 6 weeks of delivery respectively.

**Table 2:** Rates of reproductive event-associated mood symptoms/episodes among sample of postmenopausal women with bipolar disorder (*n* = 567^)

Episodes in relation to the perimenopause ^a^	
Episode (of any type) in relation to menopause, % (n)	58.5 (210/359)
*Depression in relation to menopause*^b^, *% (n)*	*21.4 (77/359)*
*(Hypo)mania in relation to menopause*^b^, *% (n)*	*8.9 (32/359)*

Premenstrual history	
Symptoms suggestive of PMS (broad definition) % (n)	51.3 (244/476)
Symptoms suggestive of PMDD (narrow definition) % (n)	22.7 (108/476)

Postpartum mood episodes (parous women only *n* = 432)	
Episode (of any polarity) within 6 weeks of delivery, % (n)	49.5 (202/408)
*Depression within 6 weeks of delivery*,* % (n)*	*26.2 (107/408)*
*Mania within 6 weeks of delivery*,* % (n)*	*19.6 (80/408)*

PMDD, premenstrual dysphoric disorder; PMS, premenstrual syndrome

^a^ excludes 208 cases where women were unsure if they had experienced episodes in relation to the perimenopause

^b^ rated where sufficient information was provided to be able to classify the polarity of the episode

^ denominators vary due to missing/unknown data

### Relationships between lifetime reproductive events

Women with a possible history of PMS symptoms were significantly more likely to have experienced mood episodes (of any type) in relation to the menopause compared to those without PMS symptoms (77% vs. 42.2%, *p* < 0.001) and episodes of depression in relation to the perimenopause (31.7% vs. 12.2%, *p* < 0.001) (Table 3). Similar significant associations were found using the narrower definition of possible PMDD: 76.4% vs. 55.5%, *p* = 0.002 (episode in relation to menopause of any type, possible PMDD vs. no PMDD) and 37.5% vs. 17.8%, *p* < 0.001 (depression in relation to menopause, possible PMDD vs. no PMDD) (Table 3). There were no significant associations found between history of possible PMS or PMDD symptoms and the occurrence of (hypo)mania in relation to the menopause (Table 3).

**Table 3:** Relationships between premenstrual symptoms and mood episodes in relation to the perimenopause

		PMS (*n* = 161)	No PMS (*n* = 147)	*p* value
Episode (of any type) in relation to perimenopause, % (n)		77.0 (124/161)	42.2 (62/147)	**< 0.001**
Depression in relation to perimenopause, % (n)		31.7 (51/161)	12.2 (18/147)	**< 0.001**
(Hypo)mania in relation to perimenopause, % (n)		9.9 (16/161)	9.5 (14/147)	0.903

		PMDD (*n* = 72)	**No PMDD** (*n* = 236)	*p* value
Episode (of any type) in relation to perimenopause, % (n)		76.4 (55/72)	55.5 (131/236)	**0.002**
Depression in relation to perimenopause, % (n)		37.5 (27/72)	17.8 (42/236)	**< 0.001**
(Hypo)mania in relation to perimenopause, % (n)		4.2 (3/72)	11.4 (27/236)	0.068

There were no significant differences in the overall rates of menopausal mood episodes (of any type) among women with and without a history of postpartum mood episodes (Table 4). However, when explored further, depressive episodes in relation to perimenopause were significantly more common among women who experienced a postpartum mood episode within 6 weeks of delivery compared to parous women who did not experience a postpartum mood episode (26.3% vs. 15.6%, *p* = 0.036). This association was stronger when only including women who experienced postpartum depression (32.8% vs. 18.1%, *p* = 0.013) (Table 4). Postpartum mania was not associated with depression in relation to the menopause. No significant associations were found between the occurrence of postpartum mood episodes and the occurrence of (hypo)mania in relation to the menopause (Table 4).

**Table 4:** Relationships between postpartum and perimenopausal episodes

	Postpartum episode (of any polarity) within 6 weeks (*n* = 133)	No postpartum episode within 6 weeks (*n* = 122)		*p* value	
Episode (of any type) in relation to perimenopause, % (n)	60.2 (80/133)	62.3 (76/122)		0.726	
Depression in relation to perimenopause, % (n)	26.3 (35/133)	15.6 (19/122)		**0.036**	
(Hypo)mania in relation to perimenopause, % (n)	6.8 (9/133)	13.1 (16/122)		0.089	

	Postpartum depression within 6 weeks (*n* = 67)	No postpartum depression within 6 weeks (*n* = 177)		*p* value	
Episode (of any type) in relation to perimenopause, % (n)	64.2 (43/67)	60.5 (107/177)		0.593	
Depression in relation to perimenopause, % (n)	32.8 (22/67)	18.1 (32/177)		**0.013**	
(Hypo)mania in relation to perimenopause, % (n)	4.5 (3/67)	11.3 (20/177)		0.104	

	Postpartum mania within 6 weeks (*n* = 59)	No postpartum mania within 6 weeks (*n* = 186)		*p* value	
Episode (of any type) in relation to perimenopause, % (n)	61.0 (36/59)	61.8 (115/186)		0.911	
Depression in relation to perimenopause, % (n)	22.0 (13/59)	22.0 (41/186)		0.999	
(Hypo)mania in relation to perimenopause, % (n)	11.9 (7/59)	9.1 (17/186)		0.540	

### Predictors of menopausal episodes after adjusting for covariates

Binary logistic analyses were carried out to identify whether a history of reported (i) PMS, (ii) depression within 6 weeks of delivery and (iii) mania within 6 weeks of delivery predicted an episode in relation to the menopause (three separate models: any episode, depression, mania). After adjusting for potential confounding variables (current age, depressive episodes per illness year, hypo(manic) episodes per illness year, parity (number of deliveries), age of onset of BD and reported age at menopause) the following reproductive events were significant predictors of episodes in relation to the perimenopause:

#### Predictors of episode (of any type) in relation to perimenopause

History of PMS was significantly associated with any type of mood episode during the perimenopause (OR 6.189, 95% CI 2.890–13.250, *p* < 0.001). Postpartum depression and postpartum mania within 6 weeks of delivery were not significant.

#### Predictors of episode of depression in relation to perimenopause

History of PMS (OR, 2.709, 95% CI 1.175–6.248, *p* = 0.019) and postpartum depression within 6 weeks of delivery (OR 2.635, 95% CI 1.119–6.208, *p* = 0.027) were significantly associated with depressive episodes during the perimenopause. Postpartum mania was not significant.

#### Predictors of (hypo)mania in relation to perimenopause

PMS, postpartum depression and postpartum mania within 6 weeks of delivery were all not significant. The same pattern of results was found when using the narrower definition of possible PMDD which predicted both an episode of any type (OR 3.014, 95% CI 1.229–7.390, *p* = 0.016) and episode of depression (OR 3.033, 95% CI 1.286–7.156, *p* = 0.011) in relation to the perimenopause.

## Discussion

To date this is the largest study to investigate relationships between illness exacerbation in relation to lifetime reproductive events among postmenopausal women with BD stratifying by episode type in the postpartum and perimenopause periods. Specifically, we found that a history of experiencing PMS and severe premenstrual symptoms (suggestive of PMDD) was associated with experiencing episodes of any type and depression during the perimenopause. Postpartum depression within 6 weeks of delivery was further associated with depression during the perimenopause. In contrast, no significant relationships were identified with (hypo)mania in relation to the perimenopause.

Although there is very limited research to date, our findings support those of Perich et al. (2017a) who found that women with BD who experienced postnatal episodes were more likely to experience worse mood symptoms menopausally. In contrast to a case series of five women with BDI which highlighted a potential link between postpartum psychosis and menopausal mood episodes (Robertson Blackmore et al. 2008), postpartum mania was not associated with the occurrence of any type of episode in relation to the menopause in our sample. The findings in our sample specifically suggest a relationship between postpartum depression and depression in relation to the menopause.

The association between premenstrual symptoms and episodes in relation to the menopause has not been found in the small number of studies that have previously explored this relationship in BD. For example, in a study by Payne et al. (2007) using a similar retrospective study design, among 197 women with BDI neither premenstrual nor postpartum mood symptoms were associated with perimenopause symptoms. The authors did not however stratify their analyses by mood symptom type during the postpartum or menopausal period. In our study we also did not find an overall association between menopausal mood episodes (of any type) and postpartum mood episodes. Significant relationships were specifically found between depression in relation to the menopause and postpartum depression. Furthermore the authors only included women with BDI in their analysis of associations between reproductive cycle-associated mood symptoms whereas the current study included women with bipolar spectrum disorder (predominantly BDI and BDII) meaning the results are not directly comparable. In the same study by Payne et al. (2007) among a larger sample of 509 women with major depressive disorder, premenstrual and postpartum mood symptoms predicted perimenopausal symptoms. Using a prospective design, Marsh et al. (2015) did not find a significant association between self-reported history of mood exacerbation premenstrually and/or during the postpartum and mood symptom severity during the perimenopause among 44 women with BD. However the different study design, definitions and measurements may account for the different findings with our study. For example Marsh et al. (2015) asked for self-reported endorsement of a history of premenstrual or postpartum mood exacerbation and did not differentiate between types of mood exacerbation in the postpartum.

Our finding of an association between reproductive life events, in particular premenstrual symptoms and postpartum depression and depression in relation to the menopause, suggest some women with BD might have a greater vulnerability to episodes of mood disturbance during reproductive events. If replicated the findings of this study have clinical implications in terms of potentially identifying women with BD who may be particularly vulnerable to episodes of depression during the perimenopause based on a previous history of severe premenstrual symptoms and postpartum depression. Providing women with information about this risk could also be incorporated into BD self-management programmes to increase awareness. Worsening of mood symptoms during reproductive events may have shared psychosocial and biological susceptibility factors. The fact that associations between reproductive events remained significant when controlling for previous number of mood episodes suggests our findings cannot be explained simply by women having an increased susceptibility to depression more generally across their lives. It has been suggested that there may be a subgroup of women with BD that have sensitivity to hormonal changes that occur during reproductive events (Teatero et al. 2014). In relation to psychiatric symptoms related to reproductive life events more generally there is growing interest in the role of allopregnanolone (ALLO), a neuroactive metabolite of progesterone and modulator of the GABA(A) receptor complex (Martinez et al. 2016; Osborne et al. 2017; Slopien et al. 2018; Hantsoo and Epperson 2020).

The strengths of the current study are that our study population was recruited from across the UK, and provided a large sample of postmenopausal women with BD. Diagnostic and rating procedures were informed by rich clinical descriptions and inter rater reliability was formally assessed and found to be high. However, as women in our study were recruited as part of a large on-going genetic epidemiology study of mood disorders within the UK it is likely our sample is not fully representative of the broader population of women with BD. Furthermore, the majority of women had a diagnosis of BDI (72.1%) which may limit the generalisability of the results. A limitation of our study is that our measure of mood episodes during the perimenopause was based on self-report, and we are unable to establish if these would meet criteria for DSM mood episodes including mixed episodes. As women were asked to self-report if they were postmenopausal we were unable to stratify our analysis further by menopausal stage for example those outlined in the Stages of Reproductive Aging Workshop (STRAW) criteria (Harlow et al. 2012). A recent systematic review and meta-analysis of 17 non-clinical cohort studies found that the perimenopausal stage (but not the post-menopausal stage) was associated with the risk of depressive symptoms compared to the pre-menopause (Badawy et al. 2024). We were however unable to separate out women who may have experienced mood episodes in the early postmenopausal stage in our sample. Due to numbers we were also unable to stratify our analysis according to whether or not women experienced the onset of BD in relation to the menopause. This would be interesting to explore in larger samples given the recent link found between the perimenopause and first onset BD (Shitomi-Jones et al. 2024). We also did not collect data on other potential confounding factors including age at menarche and medication including hormone replacement therapy (HRT).

Due to the retrospective reporting of episodes, some women with a history of premenstrual symptoms and/or episodes of postpartum depression may be more likely to attribute episodes to other reproductive life events i.e. the menopause. Among women who were able to establish the absence/presence of an episode in relation to the perimenopause, just under 60% reported experiencing an episode in relation to the menopause. This is lower than the findings of a longitudinal study among 47 women with BD which found 79% (37/47) experienced at least one mood episode during the perimenopause with 68% of women experiencing at least one depressive episode, 23% (hypo)mania episodes, and 13% episodes with both depressive and elevated mood (Marsh et al. 2008). This could suggest that the rates in our study might be an underestimate due to the method of data collection used but nevertheless our findings are similar in terms of depression being found to be the predominant mood episode over the menopausal period among women with BD. The findings of a more recent systematic review also highlighted that, among women with BD, the menopause was reported to be associated with increased symptoms overall and with depression in particular (Perich et al. 2017b). The rate of mood episodes in relation to the menopause in our BD sample is also similar to previous research among women with a history of major depressive disorder (MDD). In a follow up study of a US community-based prospective investigation of the perimenopause (Study of Women’s Health Across the Nation (SWAN: Bromberger et al. 2011), 59% of women with a history of MDD experienced an episode of MDD during midlife compared to 28% of women with no history of MDD (Bromberger et al. 2015).

A further limitation is that because women in our sample were postmenopausal, symptoms of possible PMS/PMDD were self-rated retrospectively. It was not possible in our study to confirm these ratings using prospective daily ratings which are required over at least two cycles for a diagnosis of PMDD in DSM-5. Despite premenstrual symptoms being rated retrospectively among women who were now postmenopausal, the rates of possible PMS and PMDD (52% and 23% respectively) are very similar to those found in a recent cross-sectional study among 262 Han Chinese women with BD using the same measure of PMS (58% and 21% respectively) (Liang et al. 2024). The women in the study by Liang et al. (2024) were all currently menstruating suggesting the rates found in our study may not have been significantly affected by the fact that women were now postmenopausal and reporting on past premenstrual symptoms. However, as highlighted in a recent systematic review and meta-analysis, prevalence of PMDD using a confirmed diagnosis via prospective ratings is lower compared to a provisional diagnosis based on retrospective symptom report which are likely to produce artificially high prevalence rates (Reilly et al. 2024). Despite this, the rate of possible PMDD in our sample (23%) is higher than the pooled prevalence of a provisional diagnosis of PMDD found in the general population (7.7%) (Reilly et al. 2024).

## Conclusions

Our study suggests that women with BD with a history of possible PMDD and postpartum depression are potentially at increased risk of experiencing episodes of depression in relation to the perimenopause. Although challenging given the time periods between reproductive life events, longitudinal research is needed using prospective daily ratings of premenstrual symptoms and differentiating between different menopausal stages (early/late) which was beyond the scope of our study. Findings may have implications in terms of identifying a subgroup of women with BD who may be particularly vulnerable to episodes of mood disturbance during reproductive events, which may help to develop a better understanding of the aetiology of mood disorders. Identifying women who may be particularly at risk of recurrence of mood episodes in relation to the perimenopause has clinical implications as well as for women with BD themselves. If further research confirms our findings, clinicians would potentially be able to predict the occurrence of a mood episode in relation to the menopause for each of their female BD patients based on history of postpartum mood episodes and premenstrual symptoms. This knowledge would enable clinicians to put measures in place to help educate their patients and to support them through this period of increased risk, for example, self-monitoring of mood symptoms, more frequent medication reviews and access to tailored psychoeducation and self-management programmes.
